# Comparative Extraction of Polyphenolic Co-Pigments and Proteins from Annatto (*Bixa orellana* L.) Seed By-Products

**DOI:** 10.3390/foods15111900

**Published:** 2026-05-28

**Authors:** Elsa F. Vieira, Pamela Ramires, Manuela M. Moreira, Cristina Delerue-Matos

**Affiliations:** 1 REQUIMTE/LAQV, Instituto Superior de Engenharia do Porto, Instituto Politécnico do Porto, Rua Dr. António Bernardino de Almeida 431, 4249-015 Porto, Portugal; 2 Faculdade de Filosofia, Ciências e Letras de Ribeirão Preto, Universidade de São Paulo, São Paulo 05508-220, Brazil

**Keywords:** annatto seeds by-product, bixin, phenolics, proteins, maceration, ultrasound-assisted extraction, subcritical water extraction

## Abstract

Annatto (*Bixa orellana* L.) seed by-products (ASBs), generated after industrial pigment extraction, remain a largely underexplored source of bioactive compounds, including residual carotenoids, phenolics, and proteins. This study compares three extraction strategies—maceration (ME), ultrasound-assisted extraction (UAE), and subcritical water extraction (SWE)—for the recovery of these compounds from ASB within a green processing framework. Extraction efficiency was assessed based on the yield, protein recovery, total carotenoid content (TCC), total phenolic content (TPC), antioxidant capacity (DPPH, ABTS, FRAP), and phenolic profile (HPLC-DAD). SWE at 140 °C (40 bar, 1:30 g/mL) achieved the highest extraction yields and compound recovery (proteins: 80.0 ± 3.22 mg/g extract; TCC expressed as bixin equivalents: 1.60 ± 0.07 mg/g extract; TPC: 17.96 ± 0.90 mg GAE/g extract). Phenolic profiling identified gallic and protocatechuic acids as major constituents, with significantly higher concentrations in SWE extracts. However, discrepancies between spectrophotometric and chromatographic data suggest the presence of unidentified phenolics. Cytotoxicity assays using Caco-2 cells indicated no significant effects at concentrations up to 10 µg/mL. While SWE demonstrates superior extraction performance, further studies on protein functionality, pigment stability, and bioavailability are required to validate food and nutraceutical applications. This work highlights ASB as a promising resource for circular bioeconomy strategies.

## 1. Introduction

*Bixa orellana* L. (annatto), native to the Americas and widely known as urucum or axiote, is a major natural source of carotenoid pigments, primarily bixin and norbixin, which account for 80–90% of total carotenoids and are responsible for its characteristic reddish–yellow color [1,2,3]. Annatto seeds contain significant amounts of proteins, carbohydrates, lipids, and pigments, with the composition influenced by the cultivar and environmental conditions [2,4,5]. Bixin (E160b) is approved as a food colorant and widely used in food, pharmaceutical, and cosmetic industries, with reported antioxidant and bioactive properties [2,6,7]. Beyond carotenoids, annatto also contains diverse phytochemicals, including polyphenols, flavonoids, and terpenoids, contributing to multiple biological activities [8,9,10].

Global annatto production reached approximately 30,000 tons in 2024, with Brazil as a leading producer [2,11]. Industrial pigment extraction yields a large volume of annatto seed by-product (ASB), representing over 96% of processed material and retaining proteins, carbohydrates, fibers, and residual pigments [12]. Despite its compositional richness, the integrated valorization of ASB remains underexplored, particularly regarding the simultaneous recovery of multiple bioactive fractions and protein components for food applications [13,14,15,16]. This underexploitation contrasts the increasing global interest in sustainable food systems and circular bioeconomy strategies aimed at converting agro-industrial residues into high-value functional ingredients.

Most previous studies involving annatto have primarily focused on maximizing pigment extraction from raw seeds, particularly bixin recovery, while comparatively little attention has been devoted to the integrated valorization of post-industrial ASB. Existing approaches generally target single classes of compounds (mainly carotenoids or phenolics) using solvent-intensive extraction systems and rarely considering the simultaneous recovery of multiple bioactive fractions, including proteins [3,6,8,9]. From a technological perspective, this fragmented approach limits process efficiency and reduces the economic feasibility of annatto biorefinery strategies. Scientifically, it also overlooks the possibility that proteins, phenolics, and residual carotenoids may coexist in extracts with complementary antioxidant or techno-functional properties relevant for food applications.

Conventional extraction processes rely on organic solvents or alkaline systems, which may compromise compound stability and safety [17]. To overcome these limitations, advanced techniques, including ultrasound, microwave, supercritical CO_2_, and pressurized liquid extraction, have been developed to improve efficiency and reduce solvent use [3,8,9,18,19,20,21,22,23,24,25,26,27,28]. Nevertheless, most of these studies remain focused on raw annatto seeds rather than industrial residues, and comparative assessments between extraction technologies under equivalent experimental conditions are still limited. Moreover, the selective recovery of carotenoids has often been prioritized over broader compositional valorization. While solvent-based methods can enhance pigment recovery, they may also affect stability and selectivity [29], and reported yields remain highly dependent on process conditions [6,8,9].

Subcritical water extraction (SWE) has emerged as a particularly promising green extraction strategy because water under subcritical conditions exhibits a reduced dielectric constant, enhanced diffusivity, and tunable polarity, allowing the extraction of both polar and moderately non-polar compounds without the use of organic solvents [30,31]. SWE has demonstrated high efficiency for recovering phenolics, pigments, and bioactive compounds from several agro-industrial matrices [32,33], yet its application to the simultaneous recovery of proteins, phenolics, and carotenoids from ASB has not been systematically addressed.

**Figure 1:**
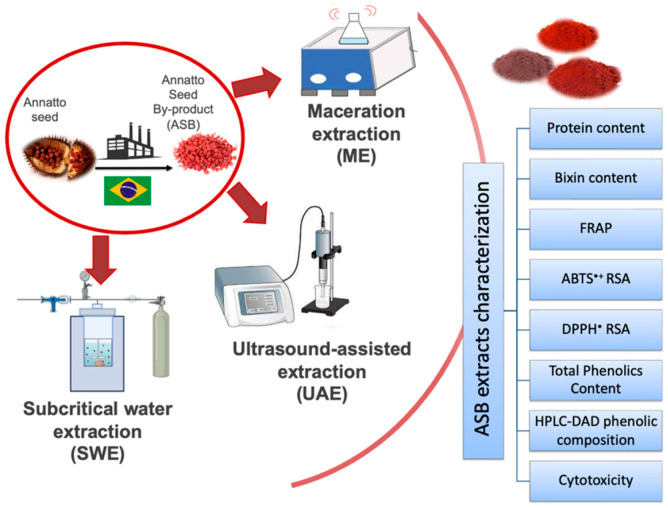
Comparative extraction of phenolic compounds, bixin and antioxidant compounds from ASB via maceration extraction (ME), ultrasound-assisted extraction (UAE), and subcritical water extraction (SWE); and characterization of promising bioactive extracts.

Therefore, the present work addresses a dual scientific and technological gap: (i) the lack of integrated strategies for the multi-component valorization of ASB, and (ii) the limited understanding of how green extraction technologies influence the simultaneous recovery of nutritionally and functionally relevant fractions from this industrial residue. In this context, maceration extraction (ME), ultrasound-assisted extraction (UAE), and SWE were comparatively evaluated regarding the extraction yield, protein recovery, total carotenoid content, antioxidant activity, and phenolic composition, together with a preliminary cytotoxicity assessment. By adopting a multi-target valorization approach, this study contributes to the development of more sustainable annatto-processing chains and supports the conversion of ASB into potentially valuable ingredients for food, nutraceutical, and circular bioeconomy applications (Figure 1).

## 2. Materials and Methods

### 2.1. Reagents and Materials

Folin–Ciocalteu phenol reagent, 2,2-diphenyl-1-picryl-hydrazyl-hydrate (DPPH), 2,2-azino-bis-3-ethylbenzothiazolin-6-sulfonic acid (ABTS), 6-hydroxy-2,5,7,8-tetramethylchroman-2-carboxylic acid (trolox), bixin, and ascorbic acid and were all purchased from Sigma Aldrich (Steinheim, Germany). Standards used in the HPLC-DAD analysis were purchased from Sigma-Aldrich (Steinheim, Germany) and Extrasynthese (Genay, France) and presented analytical purities ≥90%. The tested phenolic acids included gallic, protocatechuic, neochlorogenic, caftaric, chlorogenic, 4-*O*-caffeoylquinic, vanillic, caffeic, syringic, *p*-coumaric, ferulic, salicylic, sinapic, ellagic, and 4,5-di-*O*-caffeoylquinic acids. Flavonoid standards comprised (+)-catechin, (-)-epicatechin, rutin hydrate, quercetin-3-*O*-galactoside, quercetin-3-*O*-glucopyranoside, myricetin, and naringin. HPLC-grade methanol and formic acid were obtained from Merck (Darmstadt, Germany), and ethanol absolute was from Carlo Erba (Val de Reuil, France). Dulbecco’s Modified Eagle’s Medium (DMEM) was supplied by Invitrogen Corporation (Life Technologies, S.A., Madrid, Spain), and dimethyl sulfoxide (DMSO) was obtained from AppliChem (Darmstadt, Germany). Ultrapure water resistivity of 18.2 MΩ cm was from a Simplicity 185 water purification system (Millipore, Molsheim, France). All the spectrophotometric assays were performed using a Synergy HT W/TRF Multi Mode Microplate Reader with Gen5 2.0 software (BioTek Instruments, Winooski, VT, USA).

### 2.2. Sample Material

Annatto seed by-product (ASB) was kindly supplied by the annatto dye company New Max^®^ (São Paulo, Brazil). During the industrial process, potassium hydroxide (KOH 3% *m*/*v*) is utilized to extract pigments, while sulfuric acid is employed to precipitate them [34]. The supplied dried material with a red color (indicating the presence of bixin) was grounded (Moulinex A320, Paris, France) to an average particle size <1 mm and stored in darkness in sealed plastic bags at −18 °C until use to avoid pigment losses. The proximate chemical composition analysis of dried ASB material, including the moisture, ash, crude protein (*N* × 6.25) and crude fiber, was performed in triplicate according to AOAC (2000) Official methods 925.09, 923.03, 979.09, and 962.09, respectively [35]. The total lipid content was quantified by Bligh and Dyer (1959) [36]; the total carbohydrate content was determined by subtracting the sum of moisture, protein, fat, fiber, and ash content (%) from 100. The total pigment content was found by immersing the material in chloroform until the color present was taken completely by the solvent present. It was then quantified according to Equation (1), as followed by Balakrishnan et al. [37]:

(1) $$Total\;pigments\left(\%\right)=\frac{\left[\left(Amax+A404\right)-\left(0.256\;Amax\right)\right]}{282.6}\times \frac{100}{mass\;of\;ASB\left(g\right)}\times \frac{DV\left(mL\right)}{1000}$$

where *A_max_* and *A*404 denote the respective absorbance values of the extract at 500 nm and 404 nm, 0.256 is the factor relating the absorbances at 404 and 500 nm for bixin in chloroform, 282.6 is the absorptivity of bixin at 500 nm in chloroform, and DV is the dilution volume.

### 2.3. Extraction Procedures

ASB material was submitted to three extraction procedures: ultrasound-assisted extraction (UAE), maceration extraction (ME), and subcritical water extraction (SWE). To ensure comparability between techniques, ME and UAE were performed with identical temperatures (70 °C), extraction times (30 min), and solid-to-solvent ratios (1:30 g/mL), differing only in energy input (passive vs. acoustic cavitation). SWE was evaluated at 100–140 °C and 40 bar to assess temperature-dependent solvent behavior. All extractions were performed in triplicate using independently prepared extraction batches, and each analytical determination was subsequently conducted in triplicate.

The UAE was accomplished using a Sonic Vibracell (model VC 750, Newtown, CT, USA), using a seed-to-solvent ratio of 1:30 g/mL, temperature of 70 °C, and 30 min of extraction time. The selected time and temperature conditions were based on the highest total phenolic content (TPC) values reported for annatto seeds by [6]. Sonication was performed with a probe of 13 mm in diameter with 40% of amplitude; the power was fixed at 750 W and the frequency at 20 kHz. Five solvent compositions were tested: 100% water, 100% ethanol, 100% methanol, 20:80 (*v*/*v*) water/ethanol, and 20:80 (*v*/*v*) methanol/water.

The ME was performed in a thermostatically controlled water bath shaker (model BSC127E, C from OVAN) at 100 rpm under the same extraction conditions adopted for UAE (70 °C, 30 min, 1:30 g/mL solid-to-solvent ratio), enabling a direct comparison between conventional diffusion-based extraction and acoustic-assisted extraction.

SWE experiments were conducted in a 400 mL Parr high-pressure reactor (Series 4560 high pressure mini-reactors, Parr Instrument Company, Moline, IL, USA) coupled to a Parr Reactor Controller (Series 4848, Parr Instrument Company, Moline, IL, USA). Water extractions were performed for 30 min, at different temperatures (100, 120, and 140 °C), and two seed solvent ratios (1:10 and 1:30 g/mL). The extraction temperature was limited to 140 °C to minimize carotenoid degradation, particularly bixin thermal decomposition, as previously reported [38]. During extraction, samples were continuously stirred at 250 rpm, while pressure was maintained at 40 bar under a nitrogen atmosphere to minimize oxidative degradation. In addition, the pH of SWE extracts was monitored immediately after cooling to room temperature to evaluate potential acidification resulting from thermal hydrolysis reactions occurring under subcritical conditions.

Following extraction, UAE, ME, and SWE extracts were filtered and centrifuged (5000 rpm, 15 min, 4 °C). Ethanol-containing extracts were concentrated under reduced pressure at 45 °C using a rotary evaporator (Büchi Rotavapor R-200, Flawil, Switzerland). To minimize carotenoid degradation during solvent removal and drying, all evaporation procedures were performed under reduced light exposure and mild thermal conditions. Subsequently, extracts were freeze-dried for 48 h (Telstar Cryodos-80, Barcelona, Spain) and stored in sealed amber containers at 4 °C until analysis. The extraction yield was calculated according to Equation (2), corresponding to the ratio between the recovered extract mass and initial ASB mass.

(2) $$Extraction\;yield\;\left(\%\right)=\frac{dried\;mass\;of\;ASB\;extract\left(g\right)}{ASB\;mass\left(g\right)}\times 100$$

### 2.4. Chemical Characterization of the ASB Extracts

#### 2.4.1. Protein Content and Protein Recovery (%)

The protein content of extracts was analyzed according to the official AOAC 979.09 Kjeldahl method (6.25 nitrogen-to-protein conversion factor). The protein recovery was calculated according to Equation (3), which expresses the ratio of protein and the mass of the ASB extract.

(3) $$Protein\;recovery\;\left(\%\right)=\frac{protein\;content\;of\;ASB\;dried\;extract\left(g\right)}{dried\;mass\;of\;ASB\;extract\left(g\right)}\times 100$$

#### 2.4.2. Total Carotenoid Content (TCC) and Carotenoid Recovery (%)

The total carotenoid content (TCC), expressed as bixin equivalents, BE, was determined based on UV-VIS. The extract samples (100 mg) were diluted in 10 mL of methanol, and absorbance was measured in the UV-visible region in a spectrophotometer at 470 nm. Bixin standard solutions were prepared in methanol at different concentrations (0.1 to 3.5 mg/L) to construct the calibration curve. The analysis was done in triplicate. The carotenoid recovery (%) was calculated according to Equation (4).

(4) $$Carotenoid\left(BE\right)\;recovery(\%)=\frac{TCC\;of\;ASB\;dried\;extract(g)}{dried\;mass\;of\;ASB\;extract(g)}\times 100$$

### 2.5. In-Vitro Antioxidant and Antiradical Activity of ASB Extracts

#### 2.5.1. DPPH^•^ Radical-Scavenging Activity (DPPH-RSA)

The DPPH-RSA was determined as previously described by [30]. Absorbance was measured at 517 nm, using trolox as the standard for the calibration curve. Results were expressed as mg trolox equivalents per g dry weight (mg TE/g DW). All analyses were performed in triplicate.

#### 2.5.2. ABTS^•+^ Radical-Scavenging Activity (ABTS-RSA)

ABTS-RSA was determined according to the method described by [39]. Briefly, 100 mg of lyophilized extract was dissolved in 10 mL of absolute ethanol. Then, 20 µL of sample was mixed with 180 µL of ABTS solution and incubated in the dark at 30 °C for 10 min. Absorbance was measured at 734 nm. The ABTS working solution was adjusted to an absorbance of 1.1 ± 0.02 at 734 nm using ethanol. Results were expressed as mg ascorbic acid equivalents per g dry weight (mg AAE/g DW). All analyses were performed in triplicate.

#### 2.5.3. Ferric Reducing Antioxidant Power (FRAP)

The FRAP assay was performed according to the procedure developed by [39] using ascorbic acid as the standard, and the absorbance was measured at 593 nm at 37 °C after 10 min. The results were expressed as mg AAE/g sample DW. Assays were performed in triplicate.

### 2.6. Total Phenolic Content (TPC) of ASB Extracts

The TPC was performed applying the Folin–Ciocalteu procedure [39]. The absorbance was measured at 760 nm, and gallic acid (GA) was employed as the standard for the calibration curve. TPC was expressed as mg GAE/g sample DW. The analysis was done in triplicate.

### 2.7. HPLC-DAD Phenolic Composition Profile of ASB Extracts

The phenolic profile was characterized and quantified via HPLC-DAD, as previously described by [39]. Briefly, 50 mg of lyophilized ASB extract was resuspended in methanol:water (50:50, *v*/*v*), filtered through a 0.22 µm PTFE filter, and analyzed using a Shimadzu HPLC system equipped with a C18 reversed-phase column (250 × 4.6 mm, 5 µm) at 25 °C. A 20 µL injection volume, 1.0 mL/min flow rate, and a gradient mobile phase of methanol and water containing 0.1% formic acid were used. Elution was carried out under gradient conditions as follows: 20–26.5% A (0–13 min), maintained at 26.5% A (13–18 min), increased to 30% A (18–25 min), then progressively raised to 45% A (25–50 min), 50% A (50–60 min), 55% A (60–70 min), 70% A (70–90 min), and finally to 100% A (90–100 min). The system was held at 100% A for 5 min, followed by re-equilibration to the initial conditions (20% A) over 10 min and an additional 5 min stabilization period prior to the next injection.

Phenolic compounds were identified by comparing retention times and UV–vis spectra with pure standards. Quantification was performed at 280, 320, and 360 nm according to the maximum absorption wavelength of each compound. Analyses were carried out in triplicate, and results are expressed as the mean ± SD.

### 2.8. Cell Viability Assay of ASB Extracts

The 3-(4,5-dimethylthiazol-2-yl)-2,5-diphenyltetrazolium bromide (MTT) assay was used to evaluate the effect of the extracts on the intestinal Caco-2 cell line and to assess their potential toxicity for future nutraceutical applications. Caco-2 human colon adenocarcinoma cells (ATCC, Manassas, VA, USA) were incubated for 24 h with culture medium containing different extract concentrations (0.1, 1, 10, 100, and 1000 µg/mL). Cells from passages 42–43 were used. Cell culture conditions followed the methodology described by [31].

### 2.9. Statistical Analysis

Statistical analysis was performed using IBM SPSS STATISTICS software, version 28.0.1.0, IBM Corporation, New York, NY, USA (2021). Data were analyzed for normality and homogeneity of variances using Kolmogorov–Smirnov and Leven’s tests and then submitted to one-way ANOVA, using a Tukey’s HSD (honest significant difference) as a post hoc test. Pearson’s correlation coefficients (r) with a scale (r ≤ 0.35 is a weak (low) correlation, 0.36 ≤ r < 0.68 is a moderate correlation, 0.68 ≤ r < 0.90 is a strong (high) correlation, and r ≥ 0.90 is a very high correlation) were calculated to identify trends between total phenolic content and antioxidant activity. Statistical differences were set at *p* ≤ 0.05.

## 3. Results and Discussion

### 3.1. Proximate Composition of ASB Material

The composition of ASB showed 8.8 ± 0.2% moisture. All remaining compositional parameters are expressed on a dry weight basis (DW), corresponding to 6.2 ± 0.1% ash, 13.8 ± 0.2% proteins, 4.5 ± 0.1% lipids, 41.4 ± 1.9% carbohydrates, and 16.4 ± 0.1% dietary fiber. The protein, carbohydrate, fiber, and lipid contents were lower than the contents reported by [12], which were 17.9 ± 0.9% proteins, 49.3 ± 1.4% carbohydrates, 19.5 ± 0.6% dietary fiber, and 8.3 ± 0.6% lipids, respectively (all expressed on a DW basis). These differences could be probably attributed to the method used to extract the pigment from annatto seeds. These authors analyzed ASB resulting from pigment extraction by using soybean oil, while the ASB analyzed in this work resulted from annatto seed pigment alkaline extraction. By contract, the chemical composition of ASB was very similar to that reported by [15], whereas ASB was obtained from annatto dye extraction using the alkaline method. The authors reported that ASB contained 5.8 ± 0.1% moisture, 6.9 ± 0.02% ash, 14.2 ± 0.1% proteins, and 2.5 ± 0.1% lipids.

### 3.2. Extraction Yields in ASB Extracts

The extraction yield of ASB depends on the technique (ME, UAE, and SWE) and extraction conditions applied, namely the type of solvent, solid: solvent ratio, and temperature, as evaluated in this work. ME and UAE efficiencies were compared considering the same temperature (70 °C), 30 min of time extraction, a solid:solvent ratio of 1:30 g/mL, and the type of extractive solvent (water, ethanol, methanol, and 20:80 (*v*/*v*) aqueous mixtures of ethanol or methanol). Time and temperature conditions were set considering the maximum TPC values reported by [6] for annatto seeds. Accordingly, SWE was performed at three different temperatures (100, 120, and 140 °C) and two solid:solvent ratios (1:10 and 1:30 g/mL). The maximum temperature set was 140 °C to avoid the degradation of bixin during the extraction time, as reported by [39].

**Figure 2:**
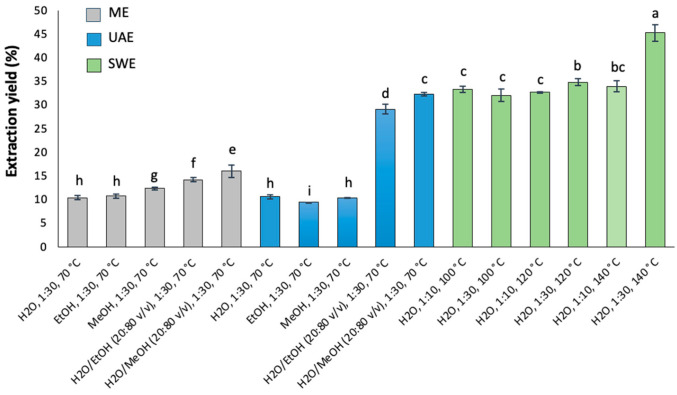
Comparison of extraction yields (%) of ASB extracts obtained using maceration extraction (ME), ultrasound-assisted extraction (UAE), and subcritical water extraction (SWE) techniques. The results were expressed as the mean ± standard deviation (n = 3). Different letters on top of bars (a–i) in the same group indicate significant differences (*p* < 0.05) between means according to Tukey’s HSD multiple range test.

As observed in Figure 2, applying ME and UAE with 20:80 (*v*/*v*) aqueous mixtures of ethanol or methanol gave significantly higher (*p* < 0.05) extraction yields than those obtained with pure solvent. UAE performed with 20:80 (*v*/*v*) water/methanol led to higher yields (32.34 ± 0.36%) when compared to ME (16.02 ± 1.34%). However, SWE reached higher extraction yields than both techniques. The SWE temperature and solid:solvent ratio parameters affected the extraction yields; SWE conducted at 140 °C with a solid:solvent ratio of 1:30 g/mL reached significant higher (*p* < 0.05) extraction yields (45.24 ± 1.77%) compared to other SWE conditions. As the temperature of subcritical water increases, its properties change continuously and the ability of dissolving the analyte increases, which allows better penetration of the matrix particles. Using a higher solid:solvent ratio also prompted higher dissolution of the ASB. Although SWE provided the highest yields due to enhanced solvent penetration and reduced viscosity at elevated temperatures, the yield alone does not reflect selectivity, and the co-extraction of non-target compounds likely contributed to higher mass recovery.

### 3.3. Protein Contents in ASB Extracts

**Figure 3:**
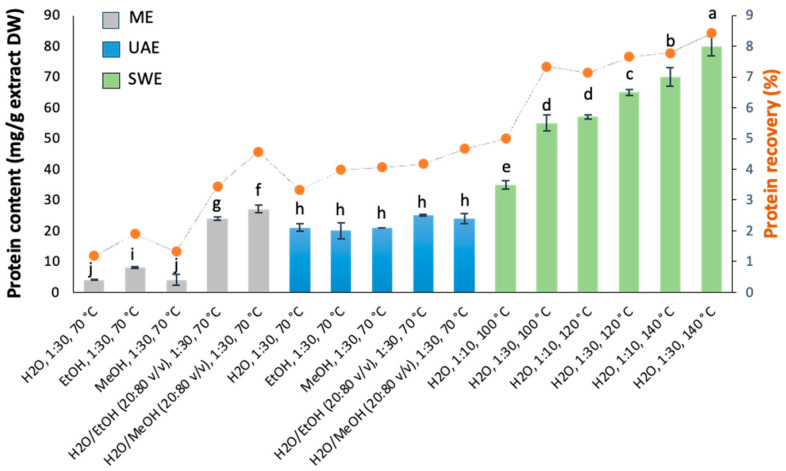
Comparison of protein content (mg/g extract DW) and protein recovery (%) of ASB extracts obtained using maceration extraction (ME), ultrasound-assisted extraction (UAE), and subcritical water extraction (SWE) techniques. The results were expressed as the mean ± standard deviation (n = 3). Different letters on top of bars (a–j) in the same group indicate significant differences (*p* < 0.05) between means according to Tukey’s HSD multiple range test.

ASB is rich in proteins, with mean contents of 13.8 ± 0.2% DW. In this work, ME, UAE, and SWE techniques were applied to ascertain the impact on ASB protein extraction. According to Figure 3, the SWE technique prompted ASB extracts with significantly (*p* < 0.05) higher protein contents (35.0–88.1 mg/g DW extract) and protein recoveries (5.0–8.4%) than ME and UAE approaches. SWE has been widely employed to extract proteins for other plant matrices [40]. Several authors recommend a temperature of 130–160 °C, as proteins are vulnerable to thermal degradation [41]. In this work, higher extraction yields of protein from ASB were obtained for SWE performed with a solid:solvent ratio of 1:30 g/mL at 140 °C, suggesting that higher temperatures and liquid-to-solid ratios favored the penetration of the extraction solvent and facilitated the mass transfer, being more effective in disrupting the ASB material and protein extraction [40].

### 3.4. TCC (Expressed in Bixin Equivalents) in ASB Extracts

**Figure 4:**
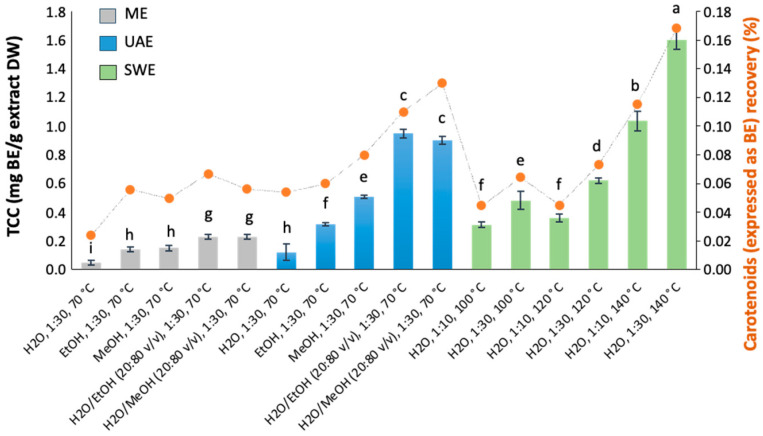
Comparison of TCC (mg BE/g extract DW) and carotenoids (expressed as BE) recovery (%) of ASB extracts obtained using maceration extraction (ME), ultrasound-assisted extraction (UAE), and subcritical water extraction (SWE) techniques. The results were expressed as the mean ± standard deviation (n = 3). Different letters on top of bars (a–i) in the same group indicate significant differences (*p* < 0.05) between means according to Tukey’s HSD multiple range test.

Demczuk and Ribani (2012) reported bixin contents in annatto seed by-products (ASB) ranging from 5 to 23 mg/g DW [42], while Silveira and Tapia-Blácido [12] described mean values of 8.7 mg/g DW. In the present work, considerably lower carotenoid recoveries were obtained regardless of the extraction technique employed, with a maximum value of 1.60 ± 0.06 mg/g DW extract observed for SWE (Figure 4). These differences may be associated with several factors, including the cultivar, climatic and agronomic conditions, pigment-extraction efficiency during industrial processing, storage conditions, particle size, and extraction parameters, such as the solvent composition, temperature, extraction time, and solid-to-solvent ratio. Importantly, the ASB used in this study originated from a previous alkaline industrial extraction process, which likely removed a substantial proportion of the native carotenoids prior to laboratory extraction.

ME was less efficient in the bixin extraction from ASB than UAE and SWE techniques, ranging from 0.05 mg/g DW sample (using 100% water, 70 °C) to 0.27 mg/g DW extract (using 20:80 *v/v* water/methanol). The bixin recovery in ME extracts was significantly (*p* < 0.05) lower, ranging from 0.02% and 0.06%. These results suggest that conventional diffusion-based extraction at a moderate temperature promotes the co-extraction of non-pigmented matrix components from the annatto seed pericarp, increasing the total extract mass and consequently reducing the relative proportion of carotenoids in the final extract [19].

Adopting UAE, the highest extraction yield of bixin (0.90 mg/g DW) was obtained with 20:80 water/methanol at 70 °C, while extraction with 100% water at 70 °C presented the lowest value, 0.12 mg/g DW sample (and lowest bixin recovery, 0.05%). Higher extraction yields of bixin were obtained with solvent mixtures of 20:80 water/ethanol or methanol, suggesting that solvent polarity was fundamental for bixin extraction, corroborating the information that bixin, a non-polar compound (carotenoid), has a greater affinity for medium-polarity extracts than in polar or non-polar solvents [6]. The combination of water with methanol or ethanol modifies solvent polarity and improves the solubilization of compounds with amphiphilic characteristics, facilitating carotenoid desorption from the residual annatto matrix. The enhancement observed under UAE treatment can be attributed to acoustic cavitation phenomena, which promote cell wall disruption, increase solvent penetration, and improve mass transfer between the matrix and extraction medium.

SWE produced the highest TCC values among all extraction approaches, with carotenoid contents ranging from 0.31 ± 0.02 mg/g DW extract (100 °C, 1:10 g/mL) to 1.60 ± 0.06 mg/g DW extract (140 °C, 1:30 g/mL). Increasing the temperature under constant pressure significantly enhanced carotenoid recovery. Under subcritical conditions, water undergoes substantial physicochemical modifications, including a reduced dielectric constant, lower viscosity, and increased diffusivity, enabling improved penetration into the lignocellulosic matrix and enhanced solubilization of moderately non-polar compounds such as carotenoids [30,31]. Elevated temperature also contributes to matrix swelling and the partial hydrolysis of structural polysaccharides, facilitating pigment release.

To our best knowledge, SWE was employed for the first time to recover bixin from ASB. Previous studies using pressurized extraction systems on annatto seeds reported maximum yields of 3.41% bixin and 1.41% norbixin under mild pressure-assisted conditions [37]. Surface morphology analyses performed in those studies demonstrated structural disruption and pigment leaching from the seed coat after extraction, supporting the hypothesis that pressure and temperature synergistically improve mass-transfer processes [37]. Nevertheless, it is important to consider that high temperatures may simultaneously promote carotenoid extraction and degradation. Bixin is thermolabile and susceptible to oxidation, isomerization, and cleavage reactions under prolonged thermal exposure [38]. Thus, the higher TCC values observed for SWE likely reflect a balance between enhanced extraction efficiency and partial thermal degradation. Since the spectrophotometric quantification employed in this work expresses the result as “bixin equivalents,” the measured absorbance may also include contributions from degradation products and structurally related carotenoid compounds.

Overall, data suggest that SWE yields more bixin than UAE and ME. This can be attributed to the use of SWE under elevated pressure, which increases the solvent’s density and diffusivity, thereby enhancing the extraction efficiency of bixin. Given that water is a non-toxic and safe solvent for bixin recovery, SWE presents a viable alternative to conventional extraction methods for obtaining bixin from ASB. However, the requirement for high-pressure equipment and elevated temperatures may increase operational costs and energy demand, aspects that should be considered in future techno-economic assessments and scale-up studies.

### 3.5. Antioxidant Activity of ASB Extracts

The industrial extraction of bixin can yield up than 90% of bixin [12]. Hence, the annatto seed residue still contains several carotenoids (bixin and norbixin, and other less important cryptoxanthin, lutein, zeaxanthin, and methylbixin), terpenoids, tocotrienols, flavonoids, and phenolic compounds responsible for the antioxidant activity [12,15,41,42], as reported in the literature (Table 1).

**Figure 5:**
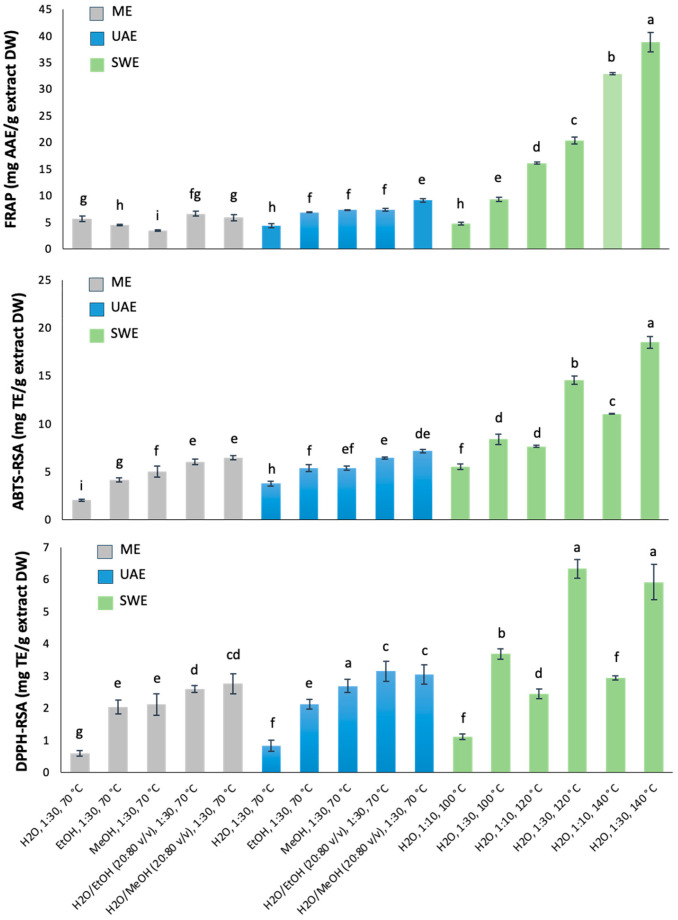
Comparison of antioxidant capacity, evaluated via FRAP, ABTS^•^-RSA, and DPPH^•^-RSA assays, of ASB extracts obtained using maceration extraction (ME), ultrasound-assisted extraction (UAE), and subcritical water extraction (SWE) techniques. The results were expressed as the mean ± standard deviation (n = 3). Different letters on top of bars (a–i) in the same group indicate significant differences (*p* < 0.05) between means according to Tukey’s HSD multiple range test.

In this work, the antioxidant activity of ASB extracts obtained via the three extraction approaches were screened by performing the in vitro FRAP, ABTS-RSA, and DPPH-RSA assays. As observed in Figure 5, the SWE technique conducted at 140 °C and using a solid-to-solvent ratio of 1:30 g/mL enabled ASB extracts with higher antioxidant capacity (29.6 ± 1.3 mg TE/g DW and 39.8 ± 2.0 mg AAE/g DW by DPPH^•^-RSA and FRAP assays, respectively) to be obtained, compared to the ME and UAE techniques. These results corroborate the significantly higher extraction yields of proteins (Figure 3) and bixin (Figure 4) observed for this treatment, suggesting that subcritical water promoted broader recovery of redox-active compounds from the ASB matrix.

The three antioxidant assays employed differ mechanistically and therefore respond differently to the compositions of the extracts. The DPPH-RSA assay primarily measures the hydrogen-atom-donation capacity of antioxidants toward a stable nitrogen-centered radical in an organic medium, favoring the detection of lipophilic antioxidants. In contrast, the ABTS-RSA assay is applicable in both aqueous and organic systems and can detect both hydrophilic and lipophilic radical scavengers. FRAP evaluates the electron-donating capacity of antioxidants through the reduction of ferric (Fe^3+^) to ferrous ions (Fe^2+^) under acidic conditions. Consequently, compounds exhibiting strong reducing power may produce elevated FRAP values even if their radical-scavenging capacity is comparatively moderate [39,43]. These mechanistic differences help explain the distinct response patterns observed among the extracts and assays.

Literature values reported for ASB extracts obtained using conventional extraction methods are generally lower than those observed for SWE extracts in this study [12,15]. Silveira and Tapia-Blacido (2018) [12] and Aguilar and Tapia-Blácido [15] reported ASB extracts via maceration with methanol and ethanol, respectively, reporting ABTS values of 2.0 and 1.78 mg trolox equivalents (TE)/g DW of ASB extract (Table 1). Both values are in the same range than values found in this work using ME (5.03 ± 0.56 and 4.13 ± 0.22 mg TE/g DW extract) and UAE techniques (5.03 ± 0.56 and 4.13 ± 0.22 mg TE/g DW extract). For all the treatments evaluated in this work, the DPPH-RSA values found in this work were in the range of 0.59 ± 0.09 mg TE/g DW extract (ME: H_2_O, 1:30, 70 °C) and of 6.33 ± 0.28 mg TE/g DW extract (SWE: H_2_O, 1:30, 120 °C), which were significantly (*p* < 0.05) lower than values reported by [15]—0.04 mg TE/g extract DW—for the ASB extract prepared using ME (EtOH, 1:20 g/mL, 8 h, 25 °C). In the present work, ME and UAE extracts showed antioxidant values within comparable ranges, whereas SWE consistently produced significantly higher responses.

### 3.6. Total Phenolic Content and Composition in ASB Extracts

TPC values reported in the literature for ASB vary considerably depending on the extraction methodology and raw material characteristics. Silveira and Tapia-Blacido (2018) [12] reported TPC values of 11.41 mg GAE/g extract in the ASB material, whereas higher values—78.8 ± 3.1 mg GAE/g extract—were reported by Aguilar and Tapia-Blácido (2023) [15]. Both studies emphasized that the species of origin, cultivation practices, and extraction methods used for annatto pigment can directly influence the final concentration of bioactive compounds in this by-product.

**Figure 6:**
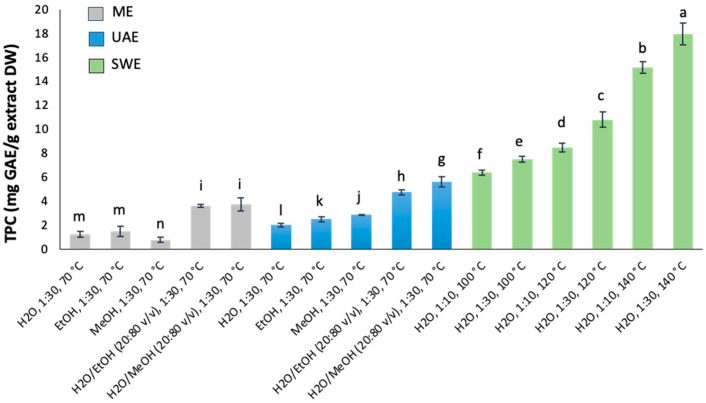
Comparison of total phenolic compounds (TPCs) of ASB extracts obtained using maceration extraction (ME), ultrasound-assisted extraction (UAE), and subcritical water extraction (SWE) techniques. The results were expressed as the mean ± standard deviation (n = 3). Different letters on top of bars (a–n) in the same group indicate significant differences (*p* < 0.05) between means according to Tukey’s HSD multiple range test.

The impact of ME, UAE, and SWE on total phenolic contents (TPCs) of ASB material evaluated in this work is shown in Figure 6.

ME results in slight rupture of cell pores, while the UAE approach utilizes cavitation effects that cause significant cell disruption and enhance intracellular mass transport [31]. For ME and UAE approaches, it was observed that a mixture of organic solvent and water is more efficient in extracting phenolic compounds than pure solvents and that, regardless of the type of alcohol used, the trend of the results was similar. For both techniques, extraction with 20:80% mixtures of water and methanol led to the highest TPC values, 3.75 ± 0.54 and 5.61 ± 0.41 mg GAE/g extract, respectively. It is well established that polar protic solvents such as ethanol and methanol can donate hydrogen to the medium, forming hydroxyl (-OH) bonds that enhance the extraction of both hydrophilic and lipophilic phenolic compounds, thereby increasing the TPC. Among these, ethanol possesses a greater hydrogen-bonding capacity than methanol. The literature also indicates that polar protic solvents with high dielectric constants in solution are more effective for polyphenol extraction than single solvents alone. Consequently, mixing water with ethanol or methanol modifies the polarity of these solvents, facilitating faster diffusion of phenolics from the ASB matrix into the extraction medium [31]. These findings are consistent with several studies reporting that phenolic compounds from annatto seeds and ASB are more soluble in aqueous ethanol solutions than in pure solvents [6].

Although UAE was more efficient in the phenolic extraction than ME, it was less efficient than SWE. In the SWE approach, using the solid:solvent ratio of 1:30 g/mL and increasing the temperature from 100 °C to 140 °C, the TPC values were 33% and 66% higher than UAE extracts prepared with 20:80% mixtures of water and methanol. This suggests that the use subcritical water as a solvent extract penetrates more easily inside the cell walls and breaks those linkages between the phenolics and cell walls increasing the phenolic compounds recovered. Despite the higher yield achieved with SWE, more expensive equipment is necessary in comparison to the requirements of ME and UAE. Nevertheless, the interpretation of TPC values in SWE extracts requires caution. The Folin–Ciocalteu assay is not entirely specific to phenolic compounds and may detect thermally generated reducing substances formed during SWE. The high temperatures employed may promote the formation of Maillard reaction products, degraded carbohydrates, furfurals, and other reducing intermediates that contribute to Folin reactivity. Therefore, the increase in TPC observed under SWE conditions likely results from a combination of the following: (i) enhanced extraction of native phenolics, (ii) liberation of bound phenolics, and (iii) generation of additional reducing compounds during thermal processing.

The TPC results are in accordance with the results previously obtained for the bixin contents (Figure 4) and antioxidant capacity (Figure 5). Pearson correlation analysis was performed among all measured responses: FRAP, ABTS-RSA, DPPH-RSA, and TPC. The stronger correlations observed between TPC and FRAP (r = 0.914, *p* ≤ 0.05) and ABTS-RSA (r = 0.839, *p* ≤ 0.05), compared to DPPH-RSA (r = 0.504, *p* ≤ 0.05), suggest that the antioxidant properties of ASB extracts are largely associated with hydrophilic reducing compounds, particularly phenolics, which aligns with the findings reported by [43] for annatto seed extracts. However, the moderate correlation with DPPH also indicates that lipophilic carotenoids such as bixin may contribute differently depending on the assay chemistry and solvent environment. Importantly, the elevated antioxidant activity observed in SWE extracts may not result exclusively from native phenolic compounds. Under subcritical conditions, thermal degradation and transformation reactions may occur, generating low-molecular-weight reducing compounds, Maillard reaction products (MRPs), and melanoidins derived from interactions between reducing sugars and amino-containing compounds naturally present in ASB. These thermally generated compounds are known to exhibit strong reducing capacity and may significantly contribute to FRAP and Folin–Ciocalteu responses. Since ASB contains both carbohydrates and proteins, SWE conditions, particularly at 140 °C, could favor the formation of such compounds. This aspect is especially relevant for interpreting TPC values determined using the Folin–Ciocalteu assay. Although widely used as a measure of total phenolics, the Folin reagent is fundamentally a redox assay and may react with multiple reducing substances besides phenolic compounds, including reducing sugars, ascorbic acid, amino acids, Maillard-derived products, and other thermally generated reductants [39]. Therefore, the higher TPC values observed for SWE extracts likely reflect not only the increased extraction of native phenolics but also the presence of additional reducing species generated during subcritical processing.

To further investigate the compounds potentially contributing to the antioxidant activity of the ASB extracts, the most promising extracts obtained using ME, UAE, and SWE were characterized via HPLC-DAD (Table 2).

SWE achieved the highest concentration of phenolic compounds (3.6 ± 0.7 mg/g DW), followed by UAE (2.1 ± 0.3 mg/g DW) and ME (1.5 ± 0.4 mg/g DW). Uuh Narvaez et al. (2023) [44] quantified 17 phenolic compounds in annatto seed extracts prepared using ME (H_2_O, solid:solvent ratio of 1:5 g/mL, 3 h, room temperature). According to these authors, the most abundant compounds included gallic acid (570.7 μg/g DW), protocatechuic acid (500.7 μg/g DW), naringenin (294.7 μg/g DW), *p*-coumaric acid (53.9 μg/g DW), and ferulic acid (30.3 μg/g DW). These compounds were also quantified in all ASB extracts. The phenolic acids were the main contributors to the phenolic profile, and flavanols represent less than 10% of all quantified compounds. The HPLC-DAD analysis also revealed that gallic acid and protocatechuic acid were the main contributors to the phenolic composition for all the analyzed extracts, with significantly (*p* < 0.05) higher mean contents found in the SWE extract (963.7 ± 98.5 and 843.7 ± 444.0 μg/g DW, respectively).

While the Pearson correlation analysis demonstrated strong relationships between TPC and antioxidant activity, particularly for FRAP and ABTS assays, reinforcing the contribution of reducing compounds to the antioxidant profile of the extracts, the HPLC-DAD quantification revealed that the sum of identified phenolics was substantially lower than the TPC values obtained via Folin analysis, particularly for SWE extracts. This discrepancy strongly suggests the presence of unidentified phenolics and/or non-phenolic reducing compounds not detected under the selected chromatographic conditions. The unidentified compounds may include polymerized phenolics, thermally transformed derivatives, Maillard-derived antioxidants, or other redox-active molecules generated during SWE. Their presence is technologically relevant because such compounds may contribute positively to antioxidant functionality and oxidative stability in food systems. At the same time, their incomplete characterization represents an important limitation for compositional interpretation. HPLC-DAD identified major phenolics; however, given the complexity of SWE extracts and the evident discrepancy between TPC and identified compounds, further characterization using LC-MS/MS and high-resolution metabolomic approaches is necessary to comprehensively identify the phenolic and thermally generated compounds present in ASB extracts.

### 3.7. Cytotoxic Effects of ASB SWE-Extracts Towards Intestinal Cells

**Figure 7:**
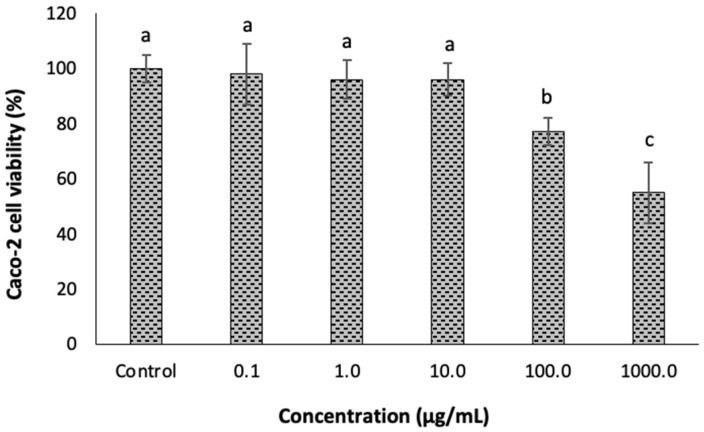
Effects of ASB-SWE extract exposure on the viability of Caco-2 cells at different concentrations (0.1–1000.0 μg/mL), measured via an MTT assay. Results are expressed as the mean ± standard deviations (n = 3). Different letters on top of bars (a–c) in the same group indicate significant differences (*p* < 0.05) between means according to Tukey’s HSD multiple range test.

The Caco-2 intestinal cell line was used as a preliminary in vitro model to evaluate the cytotoxicity of the ASB SWE-extract and to provide an initial indication of intestinal compatibility [31]. This cell line is widely employed in food and nutraceutical research to investigate intestinal absorption and cellular responses to bioactive compounds. As shown in Figure 7, the exposure of Caco-2 cells to ASB-SWE extract concentrations ranging from 0.1 to 10.0 µg/mL did not significantly affect cell viability (*p* > 0.05), with viability values remaining close to 100%. However, at higher concentrations (100.0 and 1000.0 µg/mL), cell viability decreased to 77.1% and 55.3%, respectively, demonstrating concentration-dependent cytotoxic effects. These results suggest that the ASB-SWE extract does not exhibit detectable cytotoxicity at low concentrations (10.0 μg/mL) under the specific experimental conditions evaluated. Nevertheless, the observed reduction in viability at higher concentrations indicates that the biological effects of the extract are dose-dependent and should be interpreted cautiously. Therefore, the present findings should be considered preliminary and insufficient to support broader conclusions regarding extract safety or nutraceutical applicability. Additional studies involving multiple intestinal and hepatic cell models, long-term exposure assays, oxidative stress markers, genotoxicity evaluations, and in vivo toxicological assessments are necessary to comprehensively establish the safety profile of ASB-derived extracts. Furthermore, future studies addressing gastrointestinal stability, bioaccessibility, and bioavailability will be essential to determine the physiological relevance and potential functional application of these extracts in food systems or nutraceutical formulations.

## 4. Conclusions

This study investigated the extraction of value-added compounds, namely proteins, carotenoids, and phenolic compounds, from annatto seed by-product (ASB) using three techniques: maceration (ME), ultrasound-assisted extraction (UAE), and subcritical water extraction (SWE). Among these, SWE performed at 140 °C and 40 bar with a solid-to-solvent ratio of 1:30 g/mL and yielded the highest extraction efficiency for all target compounds. HPLC-DAD analysis confirmed that SWE was particularly effective in recovering phenolic antioxidants such as catechin, caffeine, and gallic acid. Furthermore, SWE extracts exhibited no cytotoxic effects on Caco-2 cells, as demonstrated by the MTT cell viability assay, at concentrations up to 10 µg/mL. Nevertheless, several limitations must be considered: protein characterization was limited to the total content, without functional or structural analysis; pigment quantification relied on spectrophotometric methods, lacking specificity; phenolic profiling remains incomplete without mass spectrometry confirmation; and cytotoxicity results are preliminary and concentration-dependent. Therefore, while SWE shows clear potential for the sustainable valorization of ASB, further studies are required to assess protein functionality, pigment stability, bioavailability, and technological performance in food systems.

## Figures and Tables

**Table 1:** Comparison of bixin an total phenolic contents, and antioxidant activity of ASB extracts obtained in this study and previous studies.

Extraction Technique	TPC mg GAE/g Extract DW	Bixin mg/g Extract DW	DPPH^•^ RSA mg TE/g Extract DW	ABTS^•+^ RSA mg TE/g Extract DW	Ref.
Maceration	0.76–3.75	0.05–0.23	0.59–2.76	2.03–6.44	This work
Ultrasound-assisted extraction	2.01–5.61	0.12–0.95	0.83–3.15	3.79–7.17	This work
Subcritical water extraction	6.38–17.96	0.31–1.60	1.11–6.33	5.52–18.50	This work
Maceration MeOH, 1:10 g/mL, 3 h, 25 °C	11.41	8.7	NR	2.0	[12]
Maceration EtOH, 1:20 g/mL, 8 h, 25 °C	78.8	NR	0.04	1.78	[15]
Ultrasound MeOH, 1:10 g/mL followed by acetone	NR	4.99	NR	NR	[42]

Legend: ethanol (EtOH); methanol (MeOH); dry weight (DW); gallic acid equivalents (GAE); trolox equivalents (TE); total phenolics content (TPC); not reported (NR).

**Table 2:** Contents (μg/g DW) of the identified phenolic compounds in ASB extracts (via HPLC-DAD) prepared using maceration extraction (ME), ultrasound-assisted extraction (UAE), and subcritical water extraction (SWE) techniques. Results are expressed as the mean ± standard deviations (n = 3).

Compound	ME H_2_O/MeOH (20:80 *v*/*v*) 70 °C			UAE H_2_O/MeOH (20:80 *v*/*v*) 70 °C			SWE H_2_O 40 bar 140 °C		
**Phenolic** **acids**									
Gallic acid	670.5	±	21.7	689.7	±	45.8	963.7	±	98.5
Protocatechuic acid	443.8	±	13.6	698.6	±	23.8	843.7	±	44.0
Neochlorogenic acid	8.8	±	0.7	21.5	±	2.7	123.5	±	10.1
Caftaric acid	11.7	±	0.1	43.7	±	3.7	233.6	±	23.5
Chlorogenic acid	30.9	±	0.2	65.6	±	7.7	85.6	±	11.2
4-*O*-caffeyolquinic acid	39.2	±	0.6	43.8	±	5.3	81.4	±	9.8
Vanillic acid	3.2	±	0.1	14.5	±	1.7	25.7	±	4.1
Caffeic acid	2.2	±	0.1	14.4	±	2.9	54.5	±	1.3
Syringic acid	34.2	±	1.5	56.8	±	7.3	129.3	±	10.3
*p*-Coumaric acid	87.1	±	3.6	143.7	±	23.7	243.5	±	30.4
Ferulic acid	67.2	±	0.7	76.5	±	11.1	211.8	±	12.1
Salicylic acid	10.1	±	0.01	11.8	±	0.2	23.8	±	1.1
Sinapic acid	0.8	±	<0.01	11.6	±	0.9	21.6	±	1.1
Ellagic acid	11.6	±	0.1	56.7	±	2.7	64.0	±	2.5
4,5-di-*O*-Caffeoylquinic acid	0.2	±	<0.01	0.5	±	<0.01	1.7	±	<0.01
**Flavanols**									
(+)-Catechin	85.3	±	2.5	102.6	±	8.7	231.5	±	12.2
(-)-Epicatechin	12.0	±	0.8	23.6	±	3.7	163.3	±	4.5
**Flavonols**									
Rutin	<LOD			<LOQ			3.7		4.7
Quercetin-3-*O*-galactoside	<LOD			2.0		<0.01	4.6		0.7
Quercetin-3-*O*-glucopyranoside	ND			ND			12.6		2.7
Myricetin	3.8	±	0.1	4.7	±	<0.01	40.7	±	4.1
**Flavanones**									
Naringin	16.7		0.1	23.8		1.0	57.8		3.3
Σ (mg/g DW)	1.5	±	0.4	2.1	±	0.3	3.6	±	0.7
TPC (mg GAE/g DW), Figure 6	3.6	±	0.5	5.6	±	0.5	18.0	±	0.9

Legend: not detected (ND); limit of detection (LOD), limit of quantitation (LOQ); dry weight (DW), gallic acid equivalents (GAE).

## Data Availability

The original contributions presented in this study are included in the article. Further inquiries can be directed to the corresponding author.
